# Gut microbes and benign prostatic hyperplasia: the role of dysbiosis

**DOI:** 10.3389/fruro.2026.1959570

**Published:** 2026-09-02

**Authors:** Gordon Treacy, Katherine Dinan, Timothy G. Dinan

**Affiliations:** 1 Galway University Hospital, Galway, Ireland; 2 Department of Psychiatry and APC Microbiome Ireland, University College, Cork, Ireland

**Keywords:** benign prostatic hyperplasia, gut microbiota, gut–prostate axis, pro-inflammatory state, vagus nerve

## Abstract

Benign prostatic hyperplasia (BPH) is a common age-related condition characterized by stromal and epithelial proliferation within the prostate and is influenced by androgen signaling, ageing, obesity, metabolic dysfunction, and inflammation. Increasing interest has focused on whether the gut microbiota may contribute to these processes through a putative gut–prostate axis. The human gut contains a complex microbial ecosystem comprising thousands of bacterial species whose metabolites and immune-modulatory activities influence host physiology. Alterations in microbial composition and function have been associated with numerous chronic inflammatory and metabolic disorders. Emerging clinical and preclinical evidence suggests that gut dysbiosis may also be associated with prostate enlargement and lower urinary tract symptoms. Proposed mechanisms include altered production of short-chain fatty acids, impaired intestinal barrier integrity with increased lipopolysaccharide translocation, Toll-like receptor signaling, changes in tryptophan and serotonin metabolism, vagal pathways, and systemic cytokine-mediated inflammation. However, current evidence remains largely associative and mechanistic, with relatively few human studies directly linking alterations in the gut microbiota to BPH pathogenesis. This narrative review examines current evidence supporting the gut–prostate axis in BPH, evaluates potential biological mechanisms such as the vagus nerve and tryptophan, discusses dietary influences on microbiota composition, and highlights limitations in the existing literature together with priorities for future research and therapeutic development.

## 1 Introduction

This narrative review was conducted by searching PubMed, Scopus, and Google Scholar through March 2026. Search terms included “benign prostatic hyperplasia”, “BPH”, “gut microbiota”, “gut microbiome”, “gut–prostate axis”, “dysbiosis”, “short-chain fatty acids”, “lipopolysaccharide”, “Toll-like receptors”, “tryptophan”, “serotonin”, “diet”, and “prostate inflammation”. Original clinical studies, mechanistic studies, animal investigations, and relevant review articles published in English were considered. Priority was given to studies directly examining relationships between gut microbiota composition and prostate enlargement or BPH-related outcomes. Given the limited number of BPH-specific studies, evidence from related fields, including metabolic disease, systemic inflammation, and prostate inflammation, was also evaluated where relevant to the proposed biological mechanisms.

Until 20 years ago, gut microbes were considered commensal. We fed them, but they had little impact on our overall physiology. We now know that gut bacteria can produce a myriad of molecules required by organs throughout the body. The average adult has up to 1 kg of microbes in the intestine, primarily in the colon. The bulk of these microbes, found in the large intestine, consists of bacteria, though viruses, fungi, protozoa, and archaea are also present (1). The microbiome is largely defined by two bacterial phyla, Bacteroidetes and Firmicutes, with Proteobacteria, Actinobacteria, Fusobacteria, and Verrucomicrobia present at lower levels. The genera within the Firmicutes phylum include Clostridium, Lactobacillus, Bacillus, Enterococcus, and Ruminococcus. The Bacteroidetes phylum predominantly consists of the Bacteroides and Prevotella genera. The Actinobacteria phylum is dominated by the Bifidobacterium genus. For review, see Gomaa (2).

At least 1000 species of bacteria have been identified in the human gut. There is disagreement about the optimal architecture of the normal gut microbiota and, hence, a precise definition of dysbiosis. The lack of consensus is due to significant inter-individual differences in microbiota composition. However, there is agreement that greater microbial diversity in adulthood is associated with a healthy state, while the converse is also true. In addition, some genera are classified as beneficial, while others are classified as probable pathogens (3). *Bifidobacterium* and *Lactobacillus* species are generally considered beneficial and are sold in many countries as probiotics (live bacteria with health benefits) (4). In contrast, strains within the *Clostridium genus* and lipopolysaccharide (LPS)-producing taxa, such as Enterobacteriaceae, are associated with disease states (3).

The Firmicutes/Bacteroidetes ratio, which explores the relationship between the dominant phyla, has been linked to a variety of disease states. The abundance and diversity of strict anaerobes increase from childhood onward, driven by diet and environment. Despite significant individual variation in the enteric microbiota, there seems to be an optimal balance in healthy individuals. An imbalance in this ecosystem can negatively impact an individual’s well-being, increasing vulnerability to a range of diseases affecting multiple organs (5). Several factors may alter the microbiome, such as disease, diet, infection, and antibiotics, but, in general, it tends to revert to its stable, pre-disturbance diversity once the initial perturbation has resolved. This is particularly true regarding antibiotic exposure; after a week of broad-spectrum antibiotics, it may take up to 12 weeks to return to the pre-antibiotic structure (6). However, major dietary changes (e.g., switching from a carnivorous to a vegetarian diet) alter the microbiota (7). Exercise and smoking are known to produce dramatic alterations.

It has been shown that the microbiota structure of aged individuals differs from that of younger adults, and that age-related shifts in the composition and, especially, the diversity of the intestinal microbiota are linked to adverse health outcomes (8). A decrease in microbial diversity with age is associated with the onset of frailty. Notably, the volume of Bifidobacterium decreases with age.

BPH is a non-malignant growth or hyperplasia of the prostate tissue, especially common in older men with lower urinary tract symptoms (LUTS). Disease prevalence increases with advancing age (9). The histological prevalence of BPH is 50% to 60% in those in their 60s, rising to over 80% in those older than 70 years. Prostate volume increases by over 2% per annum. Risk factors such as age, genetics, and obesity have all been shown to influence the development of BPH, which is characterized by stromal and epithelial cell proliferation in the prostate transition zone, surrounding the urethra. Such a development results in urethral compression and bladder outflow obstruction, leading to LUTS, urinary retention, and, in some cases, infections due to incomplete bladder emptying. If the disease remains untreated, it can result in the development of chronic high-pressure retention with possible permanent changes to the bladder detrusor muscle. Ultimately, BPH arises due to an imbalance between prostatic cellular proliferation and apoptosis. This imbalance favors cellular proliferation, resulting in increased numbers of prostatic periurethral epithelial and stromal cells, which are visible histologically (10).

The emergence of BPH is influenced by a wide variety of factors, such as obesity, in addition to the direct hormonal effects of testosterone. Obesity has long been associated with alterations in the gut microbiota, and specific bacteria, including *Ruminococcus* and *Clostridium scindens*, are strongly correlated with testosterone levels (11). Androgens are required for BPH development, as dihydrotestosterone (DHT) stimulates cellular proliferation by directly affecting the prostatic epithelium and stroma (12). Testosterone is switched to DHT by 5-alpha-reductase 2 in prostatic stromal cells, and represents at least 90% of total intraprostatic androgen content. DHT has a direct impact on prostatic stromal and adjacent cells, thereby influencing cellular proliferation and apoptosis.

A recent review by Chen et al. (13) comprehensively summarized the role of gut microbiota in prostate inflammation and BPH. The present review differs in three respects. First, we explicitly distinguish established mechanisms underlying BPH pathogenesis from emerging microbiota-associated hypotheses. Second, we critically evaluate the strength of evidence supporting individual components of the proposed gut–prostate axis, distinguishing human observational findings from mechanistic and preclinical studies. Third, we integrate dietary, metabolic, microbial, and inflammatory pathways into a unified framework that highlights current knowledge gaps and identifies priorities for future translational research.

## 2 BPH and gut microbes

The concept of a gut–prostate axis was first introduced by Eric Yarnell and Kathy Abascal in a 2005 article (14). Since then, the concept has been expanded, especially by Chen et al. (13) in their recent review. The concept holds that gut microbes directly influence prostate function and may be relevant to the development of BPH.

Takezawa et al. (15) examined gut microbial composition in men undergoing prostate biopsy and reported a higher Firmicutes/Bacteroidetes ratio among individuals with enlarged prostates. While these findings suggest an association between gut microbial composition and prostate enlargement, they should be interpreted cautiously. The Firmicutes/Bacteroidetes ratio is a relatively crude ecological measure that can be influenced by age, body mass index, diet, medication exposure, sequencing methodology, and analytical pipelines. Bacteria such as Eisenbergiella and Ruminococcus seem to confer a protective effect against BPH, while Escherichia coli is associated with an increased risk of BPH (16). Consequently, available data support an association between prostate enlargement and altered gut microbial composition but do not establish causality. Larger longitudinal studies with detailed adjustment for confounding variables are required. In their recent detailed systematic review Xu et al. (17) provide an analysis of possible microbial alteration in BPH. Our review will focus primarily on putative mechanisms, especially the vagus nerve, short-chain fatty acids, cortisol and tryptophan.

## 3 Mechanisms by which gut microbes influence the prostate

### 3.1 Short-chain fatty acids

**Figure 1:**
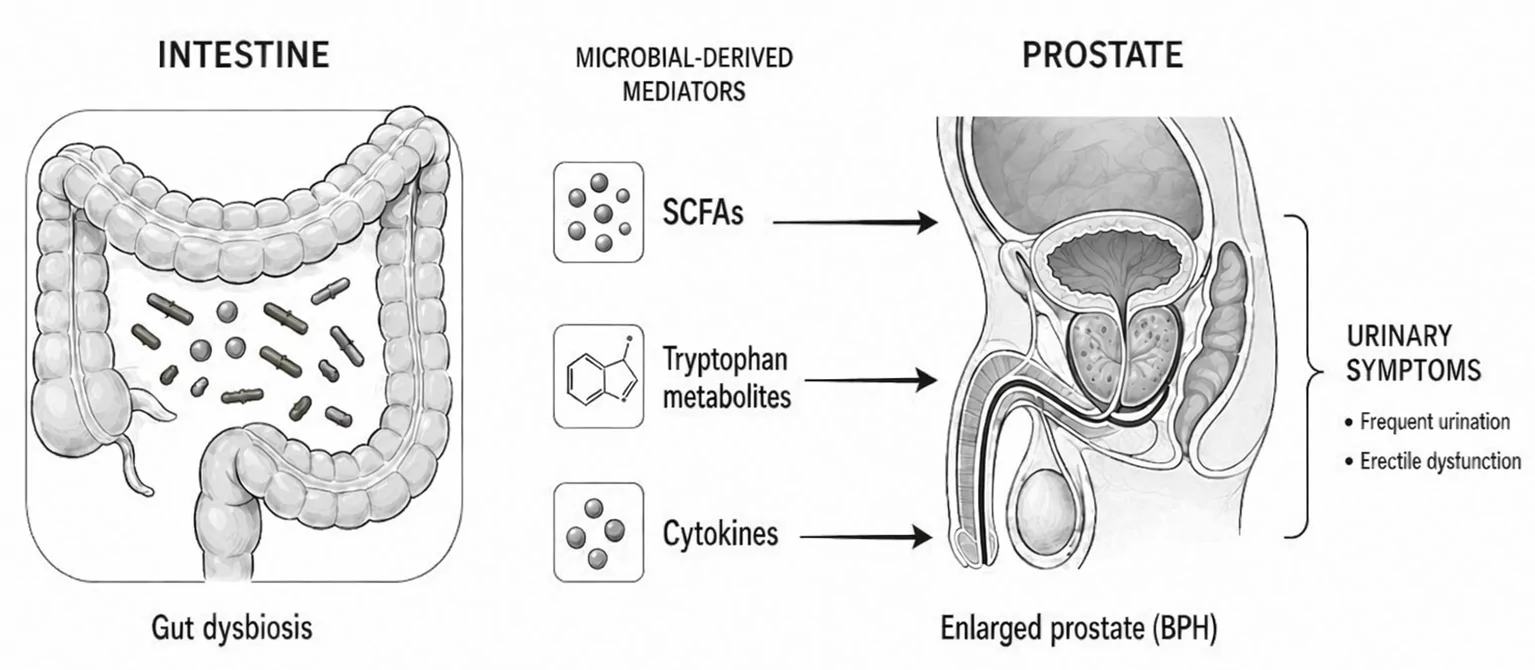
Image 1 shows the routes through which gut microbes can alter prostate function.

Gut microbes acting on fiber produce a variety of molecules, including short-chain fatty acids (SCFAs) such as butyrate, propionate, acetate and the essential amino acid tryptophan, the building block of 5-HT. SCFAs affect the G-protein-coupled receptors FFA2, FFA3, and GPR109A, as well as inhibiting histone deacetylase and thereby regulating gene expression (18). Through this mechanism, they enhance the activity of genes involved in immune activity, especially anti-inflammatory pathways. When dysbiosis occurs, SCFA production decreases, and pro-inflammatory factors increase. Chen et al. (13) also stress the importance of SCFAs in intestinal barrier function, as they increase expression of occludin and claudin genes, which play a fundamental role in gut barrier integrity. Decreased expression of these agents allows pro-inflammatory molecules from gram-negative bacteria, such as lipopolysaccharide (LPS), to enter the bloodstream. When present in the blood, the latter can induce prostatic hyperplasia (19) acting through toll-like receptor (TLR)-4 (See **Figure 1**).

### 3.2 Tryptophan

Tryptophan is derived from dietary sources and is also produced by Bifidobacteria. We showed that intake of *Bifidobacterium longum 35624* significantly increased blood tryptophan levels (20). This source of tryptophan production decreases with age as Bifidobacterium levels drop. Interestingly, centenarians tend to maintain tryptophan levels. High levels of tryptophan are associated with high levels of 5-HT in the prostate, which is associated with the inhibition of prostate growth by reducing the activity of androgen receptors (21). Studies using 5-hydroxytryptophan (5-HTP) as a potential treatment for BPH are ongoing, although the use of serotonin reuptake inhibitors (SSRIs) used to treat depression indicates a potential for urinary retention (22). The circumstances are undoubtedly complex, though in the latter case blockade of cholinergic receptors cannot be ruled out.

### 3.3 TLRs

TLRs are transmembrane proteins that function as pattern recognition receptors, providing the gateway to innate immunity. Specific receptors recognize bacterial and viral components. TLR1, 2, 4, 5, 6 and 10 detect bacterial lipids and proteins, while 3, 7, 8 and 9 detect viral and bacterial RNA/DNA (23). In the presence of dysbiosis and intestinal barrier dysfunction, LPS enters the bloodstream and binds to TLR4 receptors (24), upregulating nuclear factor-kappa B (NF-κB), which, in turn, activates the inflammasome complex and caspase-1. The latter has a profound pro-inflammatory effect.

### 3.4 Vagus nerve

The vagus nerve (cranial nerve X) is a long meandering nerve innervating most organs in the thorax and abdomen. It is now known to play a regulatory role in the pelvic area, including the prostate. We have shown that certain gut bacteria use the vagus nerve for communication purposes (25). *Lactobacillus reuteri* (JB-1), which has anti-inflammatory properties, is one such strain. It cannot function if the vagus nerve is severed. Overall, the vagus nerve helps regulate immune function probably through acetylcholine. Furthermore, patients who underwent a truncal vagotomy show a lower incidence of BPH than those who did not (26), thus providing further evidence for the impact of the vagus nerve on prostate function.

### 3.5 Gut cytokines

Conditions associated with the increased production of inflammatory cytokines raise the risk of BPH. Men with inflammatory bowel disease (IBD), both Crohn’s disease and ulcerative colitis, have higher levels of PSA and an increased risk of BPH and prostate cancer. In fact, prostate inflammation is considered an extraintestinal manifestation of IBD (27, 28). Irritable bowel syndrome (IBS) is also associated with increases in pro-inflammatory cytokines, though not to the same extent as IBD (29). Similar to IBD, patients with IBS have an increased risk of BPH. The exact reasons for this association are unclear, but it is tempting to speculate that pro-inflammatory cytokines are involved.

### 3.6 Cortisol

Cortisol is a glucocorticoid and the principal stress hormone, acting through two intracellular receptors, namely, the glucocorticoid receptor (GR) and mineralocorticoid receptor (MR)—thus regulating gene activity. Stress is recognized as exacerbating symptoms of BPH. Gut microbes play an important role in regulating the release of corticosterone in rodent. This was initially shown in germ free animals where excess corticosterone release is reduced by Bifidobacteria (30). Gut microbes have also been shown to play a key role in cortisol release in man, and microbes such as *Bifidobacterium longum* have been shown to reduce cortisol levels (31). Thus, while central factors are unlikely to play a role in the initiation of BPH, they undoubtedly exacerbate symptoms.

Most evidence supporting this mechanism comes from preclinical studies, and direct confirmation in patients with BPH remains limited (30).

## 4 Nutrition

### 4.1 Mediterranean diet

**Figure 2:**
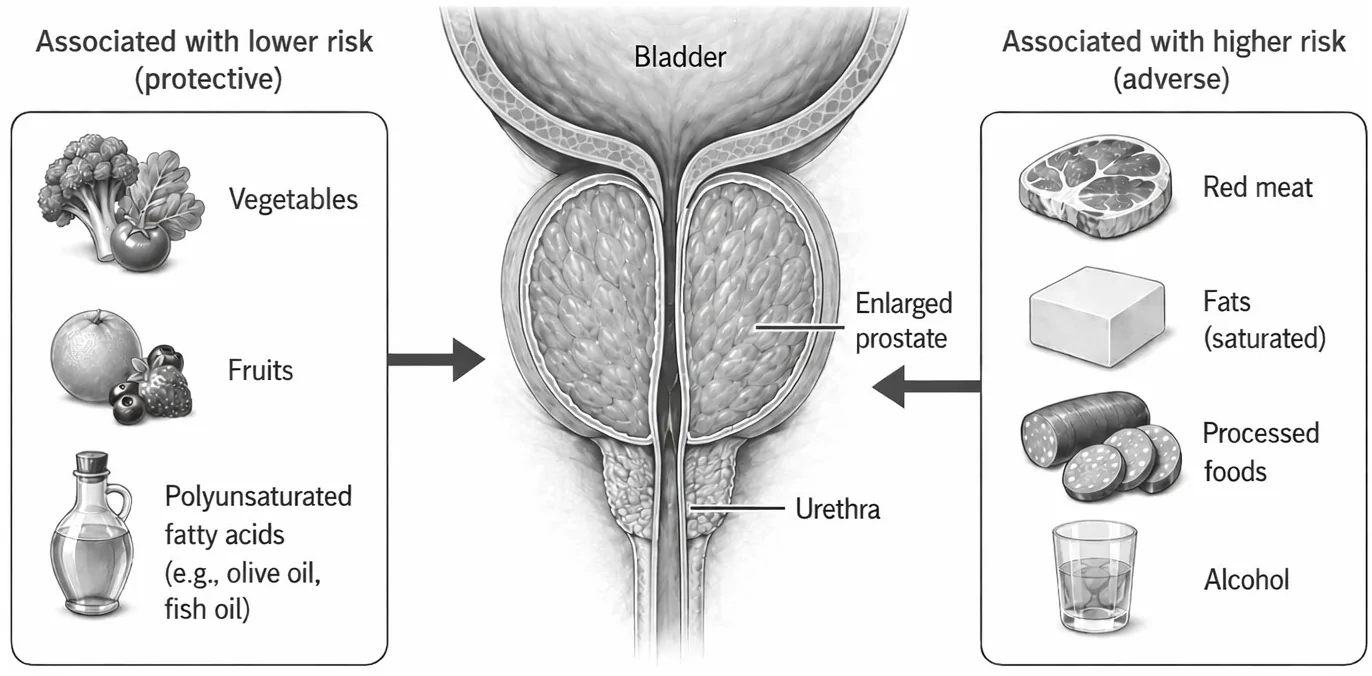
Image 2 shows the impact of nutrition on prostate function. The evidence suggests that these changes may be mediated through gut microbes.

The Mediterranean diet is rich in fruits, vegetables, grains, fermented foods, and polyunsaturated fatty acids. It is generally regarded as the optimal diet for health. A recent study demonstrated the efficacy of a Mediterranean-inspired anti-inflammatory diet in reducing inflammation in Crohn’s disease (13). De Filippis et al. (32) recently observed that Italian subjects with a high adherence to a Mediterranean diet had a greater abundance of Prevotella and short-chain fatty acids. Those with low adherence had higher urinary trimethylamine oxide (TMAO), which has associations with gut dysfunction, cardiovascular disease and BPH (See **Figure 2**).

### 4.2 Vegetarian and vegan diets

Vegetarian diets have also gained recognition as a healthy and therapeutic dietary pattern for a number of chronic diseases, while vegan diets may, in certain circumstances, confer protective benefits beyond those of vegetarian diets (33). Vegan diets may have protective effects against metabolic and inflammatory diseases such as BPH. They lead to a unique gut microbiota profile characterized by reduced levels of pathobionts. Some studies have shown that vegetarian and vegan diets significantly reduce Bacteroides fragilis counts compared to an omnivore diet. Another study comparing vegetarian to omnivore diets observed a higher ratio (%) of Bacteroidetes–Prevotella, *Bacteroides thetaiotaomicron, Clostridium clostridioforme* and *Faecalibacterium prausnitzii*, but a lower ratio (%) of the Clostridium cluster XIVa in vegetarian diets (34).

### 4.3 High-fat diet

It is well established that a high-fat diet increases the risk of BPH (35). Several studies have found that a high-fat diet leads to a decrease in Bacteroidetes and an increase in Firmicutes. Such effects are associated with increased gut permeability and peripheral inflammation.

### 4.4 Dietary proteins

Dietary proteins undergo proteolysis in the small intestinal lumen and are subsequently metabolized by the large intestinal microbiota. This triggers the generation of multiple amino acid-derived metabolites, including phenols, sulphides, ammonia, indoles, amines, and monocarboxylic acids (36). Dietary protein intake in humans has been associated with a Bacteroides enterotype. An animal-based diet in humans increases the abundance of bile-tolerant microorganisms (such as Bacteroides and Alistipes) and reduces the levels of Firmicutes that metabolize dietary plant polysaccharides. It is clear that red meat and other animal-based proteins increase the risk of BPH, while the reverse is true of plant-based proteins.

## 5 Discussion

It is clear that gut microbes play an important role in maintaining health. With unhealthy ageing, the gut microbiota loses diversity. BPH is a highly prevalent disorder which increases with age. Accumulating evidence suggests that gut microbes can alter prostate function. They can do so in a variety of ways, such as through SCFAs, tryptophan, the vagus nerve, and altered gut permeability, leading to increased LPS. This is not to downplay the importance of androgens in the pathophysiology. One major deficit in research to date on the gut-prostate axis is that much of the published work is preclinical, conducted in rodents. More intervention studies in patients with BPH are required.

Pharmacotherapy of BPH has improved little in recent years, with alpha-1 blockers such as tamsulosin and 5-alpha reductase inhibitors such as finasteride at the forefront of therapy. While research on the role of gut microbes in BPH is still in its early stages, it raises the possibility of new pharmacological treatments targeting microbes, microbial metabolites and their receptors. A recent preclinical study indicates that *B. longum* and *B. psychraeophilum* reduced oxidative species and restored androgen levels to normal physiological levels, mitigating the effect of disease (36). Similar findings have been reported for *Akkermansia muciniphila* (36–38). These studies require replication in a large patient population.
